# Blood Digestion by Trypsin-Like Serine Proteases in the Replete Lyme Disease Vector Tick, *Ixodes scapularis*

**DOI:** 10.3390/insects11030201

**Published:** 2020-03-23

**Authors:** Jeremiah Reyes, Cuauhtemoc Ayala-Chavez, Arvind Sharma, Michael Pham, Andrew B. Nuss, Monika Gulia-Nuss

**Affiliations:** 1 Department of Biochemistry and Molecular Biology, University of Nevada, Reno, Reno, NV 89557, USA; jeremiahbreyes@nevada.unr.edu (J.R.); cayalachavez@nevada.unr.edu (C.A.-C.); arvinds@unr.edu (A.S.); mpham@unr.edu (M.P.); 2 Department of Agriculture, Veterinary and Rangeland Sciences, University of Nevada, Reno, Reno, NV 89557, USA

**Keywords:** ticks, *Ixodes scapularis*, serine protease, blood digestion, trypsin

## Abstract

*Ixodes scapularis* is the major vector of Lyme disease in the Eastern United States. Each active life stage (larva, nymph, and adult) takes a blood meal either for developing and molting to the next stage (larvae and nymphs) or for oviposition (adult females). This protein-rich blood meal is the only food taken by *Ixodes* ticks and therefore efficient blood digestion is critical for survival. Studies in partially engorged ticks have shown that the initial stages of digestion are carried out by cathepsin proteases within acidic digestive cells. In this study, we investigated the potential role of serine proteases in blood digestion in replete ticks. RNA interference was used for functional analysis and a trypsin-benzoyl-D, L-arginine 4-nitoanilide assay was used to measure active trypsin levels. Hemoglobinolytic activity was determined in vitro, with or without a serine protease inhibitor. Our data suggest that trypsin levels increase significantly after repletion. Knockdown of serine proteases negatively impacted blood feeding, survival, fecundity, levels of active trypsin in the midgut, and resulted in lower hemoglobin degradation. Incubation of midgut extract with a trypsin inhibitor resulted in 65% lower hemoglobin degradation. We provide evidence of the serine proteases as digestive enzymes in fully engorged, replete females. Understanding the digestive profile of trypsin during blood meal digestion in *I. scapularis* improves our understanding of the basic biology of ticks and may lead to new methods for tick control.

## 1. Introduction

*Ixodes scapularis* is a three-host tick that requires a blood meal to complete each developmental stage, in addition to adult females needing blood for egg development [1]. The larvae and nymphs feed for 3–7 days whereas adult female feeding lasts for up to 10 days and consists of: (i) a slow feeding period up to 5–9 days post-attachment followed by (ii) rapid engorgement for 12–24 h before detachment from the host [2]. Rapid engorgement accounts for about two-thirds of the total blood meal. The tick midgut comprises a major portion of the body and consists of a ventriculus (stomach) and several pairs of highly branched ceca extending into all regions of the body. Blood digestion putatively starts in the midgut soon after ingestion and continues for several days to weeks after dropping off the host. Proteins represent about 95% of the non-water content of vertebrate blood. Consequently, hematophagous arthropods require proteases as the main enzymes in the midgut to process a blood meal [3].

A typical animal genome contains 2–4% of genes encoding for proteolytic enzymes [4]. Among these, serine proteases are the most abundant and functionally diverse group [5]. Over one-third of all known proteolytic enzymes are serine proteases. Out of a total of 233 putatively active *I. scapularis* proteases thus far identified, 63 (27%) are serine proteases [3]. Hematophagous insects such as tsetse flies, mosquitoes, and many others digest the protein-rich blood meal mainly by using trypsin-like serine proteases that have a pH optimum in the alkaline range (~8.0 pH) [6]. Protein digestion proceeds rapidly and takes place in the midgut lumen. Processing of host blood components in the tick midgut, however, appears to differ greatly from that in other hematophagous arthropods. In ticks, blood digestion is a slow process that has been shown to occur in the acidic environment of midgut intracellular vesicles (endosomes), mainly by the cathepsin-like proteases [3]. 

A multi-enzyme model for hemoglobin degradation was proposed for the European vector of Lyme disease, the castor bean tick, *I. ricinus* [7]. According to this model, the hemoglobin degradation pathway is initiated inside acidic digestive vesicles by cysteine and aspartic endopeptidases (cathepsin L, legumain, and cathepsin D), generating large peptide fragments (8–11 kDa), followed by the action of cathepsin B and C exopeptidases, generating smaller peptides (2–7 kDa). Finally, serine carboxypeptidase and leucine aminopeptidase may participate in the liberation of dipeptides and free amino acids. Other studies have suggested that the final stages of hemoglobin degradation take place both in and outside of the digestive vesicles [8]. Trypsin-like serine proteases are active at high pH [9], in contrast to the acidic-active cathepsins. Midgut homogenates of the hard tick, *I. scapularis* (formerly *I. dammini*), were shown to lyse erythrocytes from vertebrate blood at an alkaline pH, suggesting the involvement of trypsin enzymes. Ten major blood digestive proteases (cathepsin, aminopeptidase, and serine proteases) were proposed to be involved in blood digestion in *I. scapularis* [2]. The presence of four serine proteases on this list suggests previously unexplored roles of trypsin during *I. scapularis* blood ingestion. 

Most studies in tick blood digestion have focused on partially engorged females (up to 5 days on host, slow feeding phase), resulting in little information on the digestive profile beyond this stage. Therefore, to better understand the digestive enzyme profile of replete *I. scapularis*, we first characterized expression of ten proteases identified previously [2] and then measured trypsin activity in unfed, partially-fed, and post host detachment (also post-blood meal; hereafter PBM) ticks for 7 days (adults) and 28 days (larvae and nymphs) using benzoyl-D, L-arginine 4-nitroanilide (BApNA), a trypsin-specific substrate [10,11]. RNA interference was used for functional analysis of three serine protease enzymes. Our data indicate that trypsin levels increase significantly after repletion and individual knockdown of serine proteases affects the proteolytic activity of midgut extracts, suggesting that these enzymes play a major, previously unrecognized, role in blood digestion in ticks PBM. 

## 2. Materials and Methods 

### 2.1. Tick Feeding and Sample Collection

Unfed adult pathogen-free *I. scapularis* were acquired from the tick rearing facility at the Oklahoma State University, Stillwater, OK. Ticks were maintained until use in an incubator at 95% relative humidity (RH) and 20 °C.

Larvae and nymphs were fed on mice at the University of Nevada, Reno [12]. Both stages were allowed to detach naturally PBM. Detached ticks were collected daily, returned to the incubator, and harvested at the appropriate PBM intervals. Larvae and nymphs were processed at 1, 2, 3, 7, 14, 21, and 28 days PBM. Two larvae or nymphs per sample were collected in triplicate for each time point. All procedures were approved by the Institutional Animal Care and Use Committee (IACUC) at the University of Nevada, Reno (IACUC # 00682).

Adult ticks were fed on New Zealand white rabbits. Male and female adults were confined in capsules attached to the rabbit’s back using Osto-Bond skin bonding latex adhesive (Montreal Ostomy, Canada). A published protocol [13] was used with a few modifications. Briefly, feeding capsules were made out of 1.5” PVC tube with a Styrofoam lip glued for firm attachment to the rabbit’s skin. Five to six capsules were attached on each rabbit one day prior to releasing ticks. A fine nylon mesh screen was glued to the top with a small opening secured with Velcro for ease of tick removal PBM. Rabbits were fitted with a collar for the duration of tick feeding. *I. scapularis* adult females were collected 5 days post-host attachment (partially-fed), and at 1, 2, 3, 7, and 14 days PBM. Whole midguts were dissected in cold PBS buffer, washed (to remove blood), and two midguts were pooled per sample. For unfed samples, four midguts were pooled. Experiments were replicated with three biological cohorts. Once washed, midguts were immediately transferred to either a cold 1.7 mL tube containing 200 μL of Trizol or Tris-HCl-CaCl_2_ and stored at −80 °C until processed.

### 2.2. Protease Expression

Total RNA was extracted from midguts or whole ticks (larvae and nymphs) using Trizol reagent and a Zymo Direct-zZol RNA purification kit (Zymo Research, Irvine, CA, USA) with DNase treatment step. A quantity of 1 µg DNAse-treated RNA was used for cDNA synthesis, according to kit protocol (iScript, BioRad, Hercules, CA, USA). For RT-PCR, 1 µL of 1:10 diluted cDNA was used as a template in a 20 µL reaction. RT-PCR conditions for all digestive enzymes were: initial denaturation at 95 °C for 5 min, 95 °C for 30 s, 55–58 °C for 30 s (Table 1), 72 °C for 30 s, repeated for 35 cycles and a final extension at 72 °C for 10 min. 10 µL PCR productwas separated by electrophoresis on a 1.2% agarose gel along with DNA ladder (Apex DNA Ladder II; Genesee, San Diego, CA, USA) and visualized by using ethidium bromide-free dye (Amresco, Solon, OH, USA). Primer sequences for all proteases are listed in Table 1. Tubulin was used as a housekeeping control [14].

Gel band intensity was analyzed by densitometry using Image Lab 5.2.1 (Gel Doc EZ-Imager, BioRad). The DNA ladder was used as the standard for generating a linear regression model to determine PCR product abundance. The ratio of the tubulin control band intensity was used to standardize the values of the band from each gene at different time points. After calculating the ratios, the band with highest expression in each gene was set at 1 and the values were determined accordingly and plotted to accurately depict the change in expression of each gene over time.

### 2.3. Sample Preparation for BApNA Assay

Six larvae or nymphs from each PBM time point were collected in triplicates (two individuals per triplicate). Samples were sonicated in 100 µL Tris-HCl-CaCl_2_ buffer, pH 7.5, until completely homogenized and centrifuged at 12,000 rpm for 5 min at 4 °C. 10 µL supernatant from each sample was added to 90 µL of Tris-HCl-CaCl_2_ [11] followed by 200 µL of 4 mM BApNA. Serial dilutions of trypsin (Sigma) were similarly using 10 µL trypsin, 90 µL of Tris-HCl-CaCl_2_, and 200 µL of 4 mM BApNA were used to make a standard curve. Samples and standards were then placed on a shaker for 15 min at 25 °C, loaded onto a 96 well plate (100 µL sample per well), and absorbance at 405 nm was recorded on a Spectramax M5 microplate reader. For adults, midguts were dissected and pooled from two females per sample and samples were prepared and measured as above.

### 2.4. RNA Interference (RNAi) 

dsRNA was synthesized for three serine proteases (SP): SP1 (ISCW021184), SP2 (ISCW006427), and SP4 (ISCW007492). Total RNA was extracted from unfed or 1-day PBM tick midguts and cDNA synthesis as described above. Primers were designed with a T7 promoter sequence (5′-TAATACGACTCACTATAGGG-3′) on the 5′ end of both forward and reverse primers (Table 1). RT-PCR conditions were the same as mentioned above in the RT-PCR section. Gel bands were extracted using the QIAquick gel extraction kit (Qiagen, Hilden, Germany) and used as a template for dsRNA synthesis using T7 Megascript kit (Invitrogen, Carlsbad, CA, USA). Newly synthesized dsRNA was purified using phenol-chloroform and ethanol precipitated. Sixteen unfed adult female ticks per gene were injected with 1 µL of dsRNA (2 µg/µL). Injections were performed with a u-200 insulin syringe on the ventral right side between the 3rd and 4th leg of the tick. Control ticks were injected with 1 µL of RNase/DNase/Protease-free water (11 ticks). Injected ticks were immediately placed in a holding container at 95% RH and observed for 2 h for recovery before storage. Ticks were allowed to recover for 7 d before placing them on New Zealand white rabbits (see adult feeding procedure above). Detached ticks were collected daily, weighed, photographed immediately after dropping off, and stored in individual containers in the colony incubator at 20 °C and 95% RH. A batch of females from each treatment was kept for fecundity assessment. Females were observed daily for mortality and egg-laying. Egg mass was weighed once females stopped laying eggs. 

For the BApNA assay, six dsRNA-injected adult females from each of the three serine protease RNAi treatments were collected 1-day PBM. Control ticks were collected at the same time. Two midguts per sample were dissected (N = 3) and the assay was carried out as described above.

### 2.5. Hemoglobin Degradation Assay

Midguts were dissected individually from control and RNAi females collected 1-d PBM, washed, and homogenized with a pestle in 0.1 M sodium acetate, 1% CHAPS, and 2.5 mM DTT [8]. Midgut extracts were centrifuged at 16,000× *g* for 10 min at 4 °C, filtered with a 0.22 µM polyethersulfone (PES) membrane syringe filter (Olympus, Sigma-Aldrich, MO), and stored at −80 °C until used for assays. Protein concentration was measured using the BCA protein assay kit (Thermo Fisher, Waltham, MA, USA). A quantity of 0.5 µg protein extract was used to digest 10 µg of bovine hemoglobin in 25 mM Na-citrate-phosphate (pH 7.5), 2.5 mM DTT, and 25 mM NaCl. Bovine trypsin (Sigma Aldrich, St. Louis, MO, USA) was used as a control for hemoglobin digestion. Aliquots were taken out at 0, 10, 20, and 30 min post-incubation. 0.03% fluorescamine (Biotium, Fremont, CA, USA) in acetone was added to the midgut extract-hemoglobin reaction to quantify the newly formed amino-terminal ends [15]. Fluorescence was measured using a Spectramax M5 microplate reader at an excitation of 370 nm and emission of 485 nm wavelengths. Measurements were performed in triplicate. For trypsin activity inhibition, midgut extracts were pre-incubated with 0.1 mM PMSF (Research Products International, Mt Prospect, IL, USA) for 15 min at 37 °C before adding hemoglobin.

### 2.6. Statistical Analysis 

All experiments were replicated a minimum of three times with different biological cohorts. One-way ANOVA and Dunnett’s multiple comparisons were used for analysis of trypsin activity data. For the rest, a t-test with Welch’s correction was used in GraphPad (GraphPad Software, La Jolla, CA, USA). Mortality was recorded each day in control as well as three other genes of interest and then Kaplan–Meier survival analysis was performed using a Log-rank (Mantel–Cox) test to perform a pairwise comparison using control survival as reference.

## 3. Results

### 3.1. Transcript Expression

**Figure 1:**
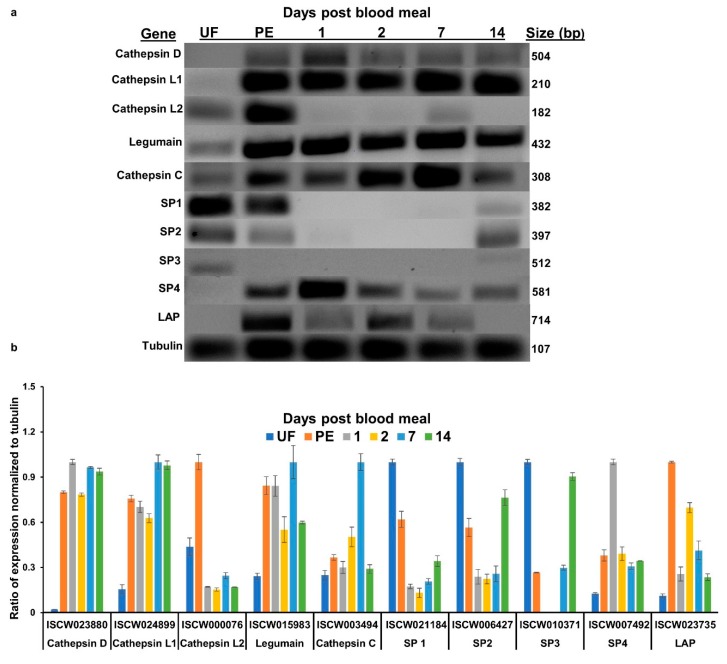
Transcript expression of proteases in the midgut of adult female *Ixodes scapularis*. (**a**) Representative RT-PCR of ten *I. scapularis* proteases, representing different protease families, at different time points during blood feeding and digestion. Total RNA was extracted from a pool of 2–4 midguts at each time point and an equal amount of cDNA was used for RT-PCR. (**b**) Densitometry of the RT-PCR gel electrophoresis images to estimate relative transcript abundance, scaled to the housekeeping gene tubulin. UF = unfed female midgut, PE = partially engorged (females were pulled from the host 5 days post-attachment), PBM = post-blood meal (fully engorged females dropped off the host). SP = serine protease; LAP = leucine aminopeptidase.

Transcripts of 10 proteases identified as the main proteolytic enzymes for hemoglobin degradation in *I. scapularis* [2] were examined in the adult female midgut. Out of these 10, four were serine proteases (ISCW021184, ISCW006427, ISCW010371, and ISCW007492), two were cathepsin L (ISCW024899 and ISCW000076), and one each were cathepsin C (ISCW03494), cathepsin D (ISCW023880), legumain (ISCW015983), and leucine aminopeptidase (ISCW023735). All sequences were confirmed by Sanger sequencing. Expression was determined at different time points: partially engorged adult females (collected 5 days post-host attachment but before rapid engorgement), and at 1, 2, 7, and 14 days PBM (fully engorged and actively digesting blood to provision developing eggs). Out of 10 genes tested, six were expressed in unfed samples whereas four genes [ISCW023880 (cathepsin D), ISCW024899 (cathepsin L), ISCW007492 (serine protease), and ISCW023735 (leucine aminopeptidase)] were only expressed during feeding or PBM (Figure 1a,b). Cathepsin D expression was highest at 1-day PBM and decreased afterward. One cathepsin L paralog (ISCW024899) expressed in all blood-fed stages tested, from partially engorged to 14 days PBM, and the other cathepsin L (ISCW000076) expressed in unfed and partially fed females with almost no expression in fully engorged females. Legumain was expressed at low levels in unfed females, then expression increased during and after feeding. Cathepsin C also expressed at low levels in unfed females and expression was higher during and after a blood meal; peak expression was at 2 and 7 days PBM and decreased at 14 days PBM. Serine proteases 1 and 2 (SP 1 and 2; ISCW021184 and ISCW006427) had a similar expression pattern in unfed and partially fed females and at 14 days PBM. Serine protease 3 (SP3; ISCW010371) had low expression in unfed and 14 days PBM samples. Serine protease 4 (SP4; ISCW007492) was not detected in unfed females, but expression was detected during and after blood-feeding with the highest expression at 1-day PBM. Leucine aminopeptidase expression was not detected in unfed females and peak expression was in partially engorged females. Expression decreased afterward and no expression was detected at 14 days PBM (Figure 1a,b). 

### 3.2. Active Trypsin in Tick life stages

**Figure 2:**
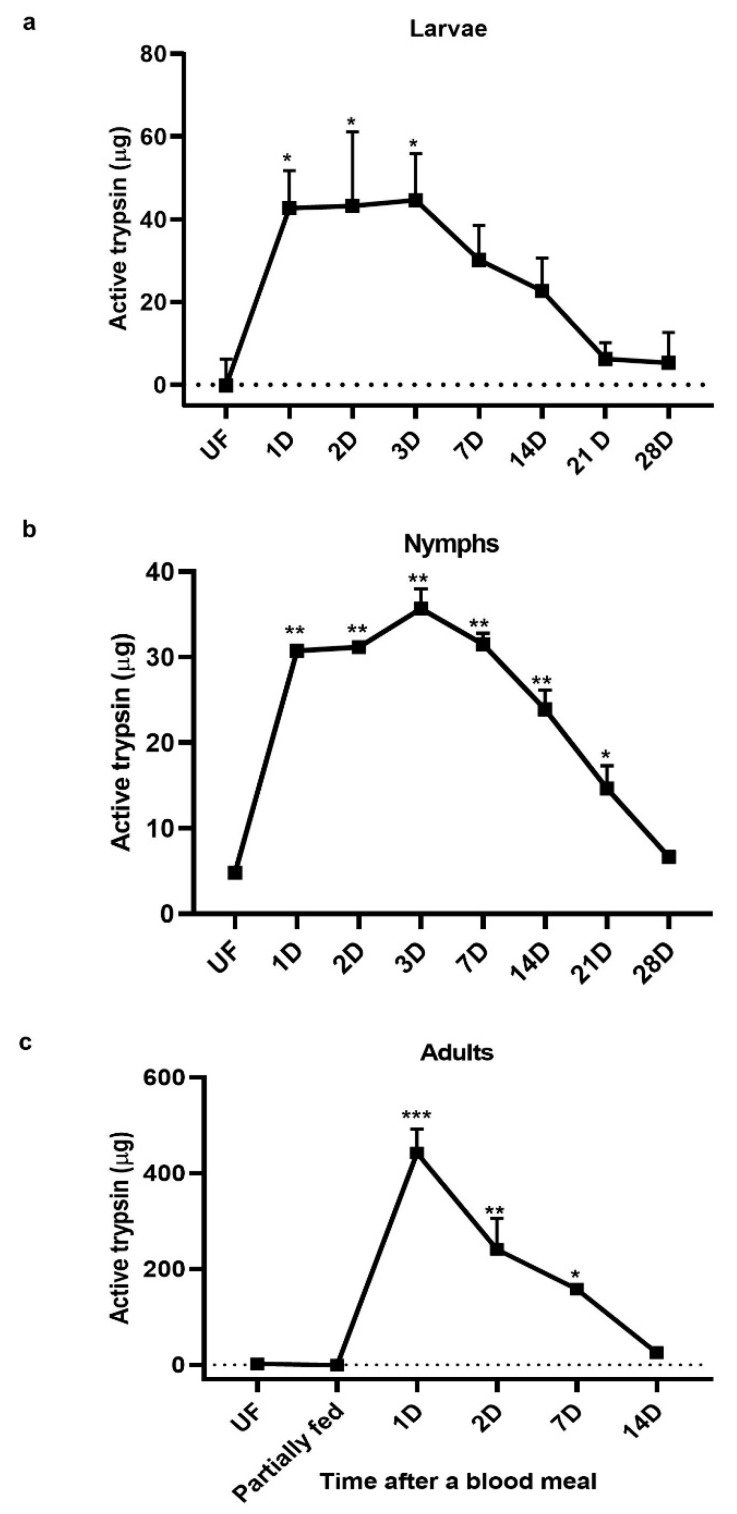
BApNA assay of trypsin activity in *I. scapularis* developmental stages. (**a**) Larvae and (**b**) Nymphs were collected before and after feeding on a mouse and whole bodies of larvae or nymphs were homogenized for use in the BApNA assay to estimate trypsin activity. (**c**) Adult females were collected before, during (5 days post-attachment, partially engorged), or at intervals after feeding on a rabbit. Dissected midguts were homogenized for use in the BApNA assay. One-way ANOVA and Dunnett’s multiple comparison were used for statistical analysis. * = 0.01; ** = 0.001; *** = 0.0001

Trypsin activity increased significantly in larvae after a blood meal (Figure 2a). The peak trypsin activity period was 1–3 days PBM and levels decreased gradually afterward to nearly unfed levels at 21–28 days PBM (Figure 2a). In our colony, larvae begin molting into nymphs within 3–4 weeks; therefore, the 28-day time-point coincides with molting. In nymphs, a similar pattern was observed. Trypsin levels increased after a blood meal and were highest at 3 days PBM. Subsequently, levels decreased and by 28 days trypsin levels were similar to those in unfed nymphs (Figure 2b). 

In adult midguts, no trypsin activity was detected in unfed or partially fed ticks. Trypsin activity was highest after drop off from the host (1-day PBM) and decreased gradually. By 14 days PBM, trypsin activity returned to the unfed levels (Figure 2c). Under our rearing conditions, ticks start laying eggs 7–10 days PBM; therefore, most blood digestion occurs during the first two weeks PBM.

### 3.3. Effect of Serine Proteases RNAi on Tick Blood-Feeding and Physiology

**Figure 3:**
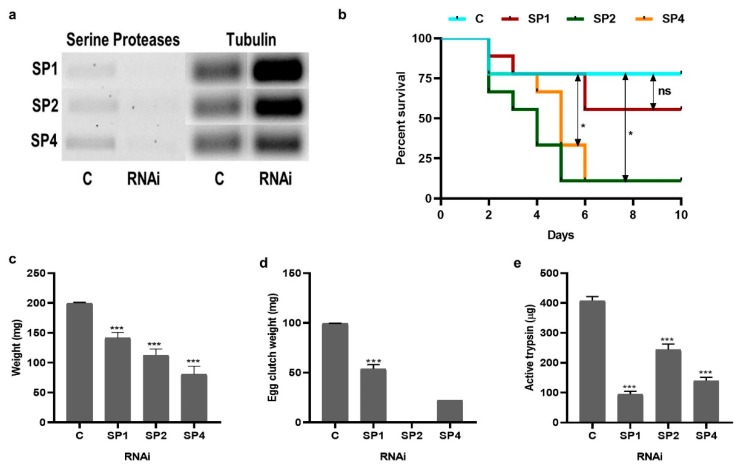
Effect of serine protease knockdown on adult female *I. scapularis* feeding, blood digestion, survival, and reproduction. (**a**) Representative RT-PCR of three *I. scapularis* serine proteases demonstrating knockdown. (**b**) Percent mortality during blood-feeding, until all ticks dropped off. (**c**) Wet weight of ticks measured immediately after dropping off the host. (**d**) Egg clutch weight, as determined after females stopped laying eggs for 2 consecutive days. (**e**) Active trypsin levels measured by the BApNA assay in midguts dissected from replete females, 1-day PBM. C: control ticks injected with water, RNAi: dsRNA injected ticks. SP: serine protease, SP1 = ISCW021184, SP2 = ISCW006427, and SP4 = ISCW007492. An unpaired t-test with Welch’s correction was used for comparing control and a treatment (SP1, SP2 and SP4) using Graphpad Prism v8. *** *p* < 0.0001.

RNAi knockdown was successful for three serine proteases (SP1, SP2, and SP4) and knockdown persisted until at least 2 days PBM (a total of 15 days) (Figure 3a). Knockdown of serine proteases by RNAi increased tick mortality. Approximately 10% of ticks died in all treatments during the recovery period post-injection. Once attached to the host, mortality was significantly higher in RNAi ticks compared to the controls. Seventy-seven percent of control ticks survived and fed to repletion in comparison to SP1 (55%), SP2 (11%) and SP4 (11%) RNAi (Figure 3b).

Most mortality occurred between 2–6 days post-attachment (Figure 3b). Serine protease knockdown also resulted in a significantly lower volume of blood ingested, as indicated by engorgement weight. Control females weighed an average of 200 mg whereas SP1 knockdown weighed 140 mg (29% reduction), SP2 knockdown weighed 110 mg (45% reduction), and SP4 knockdown weighed 78 mg (61% reduction) (Figure 3c). Fecundity was also reduced, as measured by egg mass weight. Egg clutch weight in controls was, on average, 100 mg, but was only 54 mg in SP1 and 22 mg in SP4. SP2 RNAi females did not produce any eggs (Figure 3d). Significantly less overall trypsin activity was detected in the midgut of RNAi females compared to controls at day 1 PBM (peak trypsin activity). Control ticks had an average of 400 µg trypsin compared to 95 (76% reduction), 240 (40% reduction), and 140 µg (65% reduction) in SP1, SP2, and SP4 RNAi females, respectively (Figure 3e).

### 3.4. Hemoglobin Degradation by Tick Midgut Extract 

**Figure 4:**
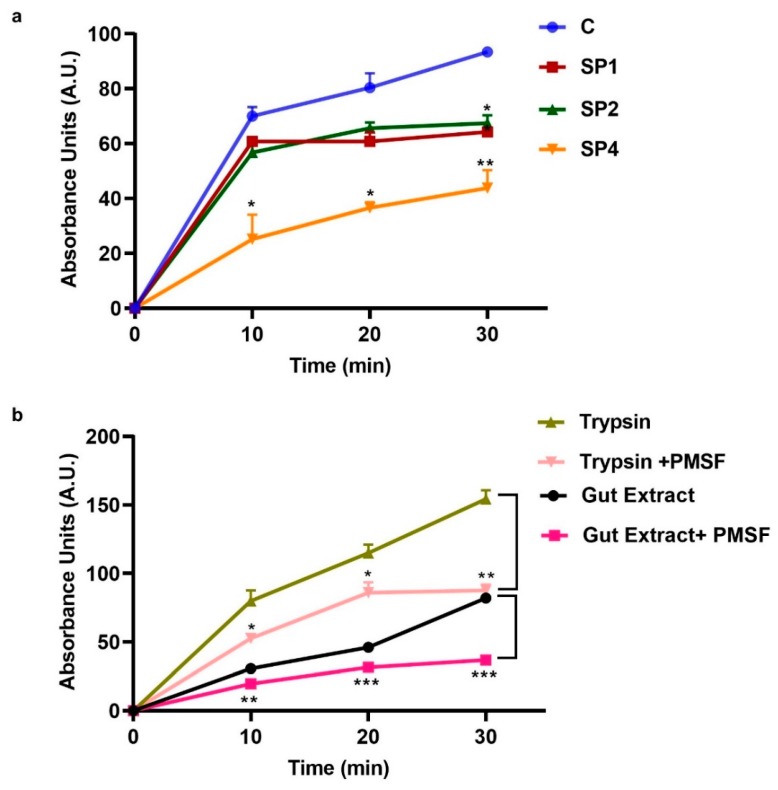
Hemoglobin degradation by *I. scapularis* midgut extract. (**a**) Effect of serine proteases knockdown on hemoglobin degradation. Homogenized midgut extract from fully engorged (1-day PBM) Control (water-injected) or SP1, SP2, and SP4 knockdown ticks was incubated with bovine hemoglobin and degradation was estimated over time by absorbance of fluorescamine (mean ± standard deviation, normalized to fluorescence intensity at 0 min). (**b**) Hemolytic activity of the tick midgut extract was significantly inhibited by pre-incubation with the serine protease-specific inhibitor PMSF. Control hemolytic reaction with bovine trypsin were similarly inhibited by PMSF. Significance was calculated separately for trypsin and trypsin + inhibitor; and gut extract and gut extract + inhibitor. An unpaired t-test with Welch’s correction with 95% confidence interval was used. * *p* < 0.05; ** *p* < 0.01, *** *p* < 0.0001.

Bovine hemoglobin incubated with tick midgut extracts at 7.5 pH resulted in free amino-terminal ends indicative of hemoglobin digestion by the midgut extract. Serine protease RNAi decreased this activity, further suggesting that these proteases are involved in hemoglobin degradation and therefore blood digestion. SP1 and SP2 knockdown resulted in significantly lower hemolytic activity compared to the control at 30 min, with a 29% and 25% reduction in hemoglobin breakdown activity (fluorescent activity), respectively (Figure 4a). SP4 knockdown had the greatest effect on hemoglobin breakdown and was significantly lower than the control throughout the assay, with a 52% reduction in midgut extract hemolytic activity at 30 min incubation (Figure 4a).

To confirm the trypsin-like protease activity in the midgut tissue extract of fully engorged *I. scapularis* females, we incubated midgut extract from water-injected ticks with a trypsin inhibitor, PMSF, prior to addition of hemoglobin. Midgut extract without PMSF incubation was used as a control. Incubation with PMSF inhibited the midgut extract activity by 55% (Figure 4b). The hemoglobin degradation activity was significantly lower in PMSF incubated samples starting at 10 min, but was even more evident at 30 min as there was no increase in activity with time compared to the non-PMSF midgut sample (Figure 4b).

## 4. Discussion

In the present work, we examined *I. scapularis* midgut protease expression including four trypsin-like serine protease transcripts. These serine proteases had a different temporal expression: two were expressed in unfed and partially engorged females, one only in unfed, and one in all blood-feeding stages tested but not in unfed midguts (Figure 1a). Since transcript abundance is not a direct measure of enzyme activity and higher transcript abundance does not always result in translation, we measured active trypsin levels. The trypsin assay showed that unfed *I. scapularis* midguts did not have trypsin activity (Figure 2c). However, trypsin activity increases after detachment from the vertebrate host and peaks at 1–3 days PBM in all life stages (Figure 2a–c). Trypsin cleaves p-nitroanliline off BApNA yielding a yellow substrate that was measured at 405 nm. This is a trypsin-specific reaction that does not occur with cathepsins or chymotrypsin [16]. In *Aedes aegypti* mosquitoes, induction of trypsin biosynthesis after the blood meal is a two-phase process. The first phase of trypsin biosynthesis involves initiation of translation of an mRNA transcript that is already present, producing early trypsin. The second phase, 7–9 h PBM, is activated by the synthesis of a new mRNA transcript that codes for late trypsin [17]. Our transcript and enzyme activity data suggest that, similar to mosquito early trypsin, the mRNA for *I. scapularis* trypsin is present in unfed ticks.

**Figure 5:**
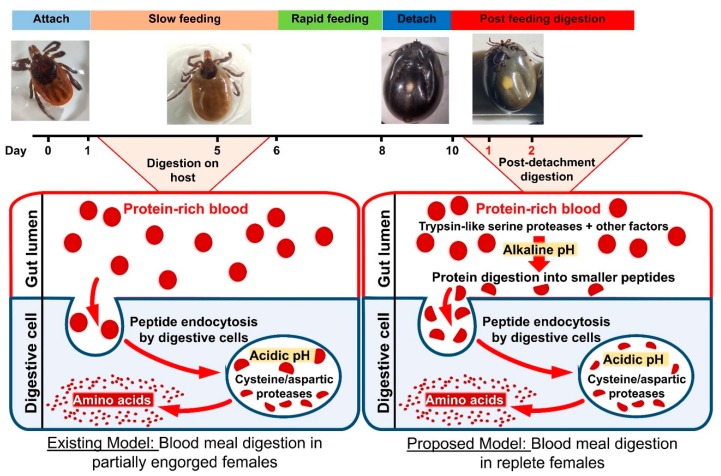
Revised model of blood digestion in replete females. Proposed model suggesting that in replete females (1–2 days PBM), ingested blood proteins are digested by the trypsin-like serine protease enzymes.

Most previous studies have focused on the mechanism of hemoglobin degradation in ticks during early stages of feeding and suggest that when ticks are actively feeding on the host, the main peptidases for hemoglobin digestion are: (1) clan CA cathepsins B, C, and L; (2) clan CD asparaginyl endopeptidase (legumain); and (3) clan AA cathepsin D. Other activities detected were attributed to monopeptidases, namely a serine carboxypeptidase and a leucine metallo-aminopeptidase, within midgut digestive vesicles [18]. Previous studies have also suggested hemoglobin receptor-mediated endocytosis [6,18] occurs in the digestive vesicles. Digestive vesicles then lead to further breakdown of these peptides by creating an acidic environment suitable for cathepsin and legumain activity. However, in this study, we investigated blood digestion after repletion and off host, a relatively unexplored blood digestion phase in ticks. Our data strongly suggest that serine proteases are involved in blood digestion in the PBM phase. Midgut extracts lysed hemoglobin in vitro and pre-incubation with trypsin inhibitor reduced this hemolysis activity (Figure 4a,b). The knockdown of three serine proteases individually resulted in lower levels of active trypsin in the BApNA assay (Figure 3e). Serine protease knockdown also resulted in reduced hemoglobin degradation activity in vitro (Figure 4a). Other studies in replete ticks have also suggested that trypsin proteases might be involved in blood digestion. Ribeiro [19] showed that midgut homogenates of *I. scapularis*, (formerly *I. dammini*) lysed erythrocytes from rabbits, rats, hamsters, and guinea pigs. The midgut homogenate activity was optimal at an alkaline pH (pH 7.5–8.5), suggestive of trypsin-like serine proteases. This activity was not detected in unfed ticks as well as ticks attached for up to 2 days to a host, but rather increased during the latter phase of feeding. It was hypothesized that this initial activity helped process the blood meal by releasing the contents of erythrocytes for further enzymatic hydrolysis, possibly in the digestive vesicles [19]. Two serine proteases in *Haemaphysalis longicornis* ticks were identified and characterized, and expression of both serine proteases was induced by blood-feeding [20]. In another study, two genes encoding trypsin-like serine proteases, HlSP2 and HlSP3, in *H. longicornis* were also proposed to be involved in blood digestion [21]. One of these HISP genes was further characterized and was found to be secreted in the midgut lumen [22]. Disruption of HlSP-specific mRNA by RNAi prevented degradation of host erythrocyte membranes, indicating that HlSP plays a crucial role in hemolysis in the midgut of ticks [22]. Hemolysin-like material was also demonstrated in the midgut lumen of ixodid ticks by immuno-localization [22,23]. An RNAseq study comparing blood-fed and serum-fed *I. ricinus* midgut transcriptomes showed that the number of genes encoding serine proteases were markedly up-regulated in the late stage of feeding [24] and the possibility of active serine proteases during the off-host stage of blood digestion was suggested. Given these data in other tick species and our results in *I. scapularis*, we propose a modified model of blood digestion in ticks (Figure 5). We suggest that the existing model of blood digestion, which occurs in digestive cells by cathepsins and aminopeptidases, applies during the early digestive phase when the tick is still feeding, whereas in replete females, a revised model is warranted which includes trypsin-like serine proteases that are important for hemolysis and initial degradation of blood proteins and that digestion may take place in both the midgut lumen and digestive vesicles.

A remarkable property of certain insect midguts is a very high luminal pH, especially in lepidopteran larvae (pH 9–12). In contrast, the midgut of the yellow fever mosquito, *Ae. aegypti*, is acidic (pH 6.0) before a blood meal, which then increases to an alkaline range (pH 7.5) after a blood meal [25]. The pH of the mite midguts also strongly affects their digestive processes. For instance, the midgut contents of acaridid mites ranged from pH 4 to 7 [26]. *I. scapularis/dammini* midgut homogenate derived from females in the rapid feeding phase had high proteolytic activity at pH 7.5–8.5 [19]. Both BApNA and hemoglobin degradation assays in this study were carried out at pH 7.5, suggesting that these serine proteases are active at an alkaline pH and provide indirect evidence of an alkaline midgut lumen environment. We attempted to measure pH by homogenizing midguts and using a universal pH paper; our results suggested an alkaline pH, but a refined method of measurement is needed (data not shown). For instance, the pH in midguts of 12 species of the stored product and house dust mites was determined based on the color changes of pH indicators fed to the organisms and looking at pH change microscopically. Unfortunately, this is not feasible with ticks due to the dark cuticle and blood meal coloration. However, microelectrodes are frequently used to determine midgut pH in insects [27,28,29,30], and we plan to utilize these in future experiments. 

The knockdown of three serine proteases resulted in ingestion of lower blood volume (Figure 3b) that correlates with lower fecundity (Figure 3d). In *H. longicornis*, knockdown of three serine proteases, HISP1, 2, and 3, also resulted in a significant reduction in the body weight compared to control group [21]. In contrast, *I. ricinus* Cathepsin D knockdown had no effect on mortality, weight, oviposition, and larvae hatching [31] but knockdown of *I. ricinus* cathepsin L1 (IrCL1) resulted in decreased weight gain in partially engorged females injected with IrCL1 dsRNA relative to the controls [32]. A cysteine protease, longipain, knockdown in *H. longicornis* also resulted in significantly lower body weight than that of the control ticks [33]. High mortality in SP2 and SP4 knockdown females’ post-attachment (Figure 3c), combined with reduced feeding, suggest additional roles in *I. scapularis* physiology that need to be further investigated. 

Here, we provide direct evidence of serine proteases as active digestive enzymes that can break down blood proteins in replete ticks. Future experiments will include the use of recombinant *I. scapularis* serine proteases for blood protein digestion assays in vitro. This initial exploration examined the most prominent *I. scapularis* digestive enzymes, which only included 3 out of a putative 63 serine proteases present in the genome [3]. In future studies, a more expansive screen will yield additional information on the dynamics of *I. scapularis* enzymes important in blood digestion.

## 5. Conclusions

Most studies in ticks suggest that blood digestion occurs in the acidic environment of midgut vesicles by the cathepsin-like peptidases in partially engorged ticks. In this study, we show that the trypsin-like proteases may play a significant role in blood protein breakdown later during feeding, in fully engorged, replete ticks. One of the major advantages of characterizing the serine proteases that regulate blood digestion is that they are generally secreted into the extracellular environment and hence they are likely to be exposed to host antibodies [29,30], making them suitable for anti-tick vaccine candidates.

## Figures and Tables

**Table 1:** A list of primer pairs and the annealing temperature used for each primer pair.

Gene Name and Accession Number	Primer Pair Sequence	Annealing Temp °C
Cathepsin D (Aspartic) ISCW023880/ XM_002416473.1		
IscapCathDFwd	CCCTTCCGTGTGGTGTTTG	55
IscapCathDRev	AGTAGCCCTTGGTTGAGACAG	55
Cathepsin L (Cysteine) ISCW024899/ XM_002416305.1		
IscapCathLFwd	GACTTCCAGATGTACCAGGGC	55
IscapCathLRev	GAAGGATGCGGAAGTAGCCG	55
Cathepsin L (Cysteine) ISCW000076/ XM_002404428.1		
IscapCathL2Fwd	AAGTGGCCCCACTGCAACTC	55
IscapCathL2Rev	TTACCCGTAACCGCAGGAATG	55
Cathepsin C (Cysteine) ISCW03494/ XM_002400742.1		
IscapCathCFwd	CGTTAACTACGTGTCCCCTG	57
IscapCathCRev	TAGTTGCCGACGTAATGCC	57
Legumain (Apartic) ISCW015983/ XM_002402043.1		
IscapLegumainFwd	CCCCTGGAGTGGTCATCAAC	55
IscapLegumainRev	TAAGTGTTTCGGAGGGCGTC	55
Serine Protease 1 ISCW021184/ XM_002405400.1		
IscapSP1Fwd	AGCCTAATCAATCAAGGGCG	58
IscapSP1Rev	GACCAGTTTAGGGATGCGAG	58
Serine Protease 2 ISCW006427/ XM_002435219.1		
IscapSP2Fwd	ATCCACGTTGGGAACCTTTC	58
IscapSP2Rev	CAATGGTCAAACGCCTTTCC	58
Serine Protease 3 ISCW010371/ XM_002402819.1		
IscapSP3Fwd	TCTACGAGTTCCTGGGACAG	58
IscapSP3Rev	GGACCAGGGAATAATCGTCG	58
Serine Protease 4 ISCW007492/ XM_002404245.1		
IscapSP4Fwd	GCTTCGTCGAAAAAGCTCAC	58
IscapSP4FRev	CAACTCTCGGCGATCTCTTC	58
Leucine Aminopeptidase ISCW023735/ XM_002416067.1		
IscapLAPFwd	ACGCCCATTCTCTCACCAAG	55
IscapLAPRev	TTCGGACCCACTGCATTCTC	55
Tubulin ISCW005137/ XM_002402966.1		
IscapB-TubFwd	TGAATGACCTGGTGTCCGAG	55–58
IscapB-TubRev	GCAAAGCTGTTCAAGCCTCT	55–58
